# Parallels between bipolar disorder and *ATP1A3*-related diseases: a window into the investigation of lithium for alternating hemiplegia of childhood

**DOI:** 10.1186/s13023-026-04235-3

**Published:** 2026-02-03

**Authors:** Jacqueline Alves Leite, Leandro Augusto de Oliveira Barbosa, Regina L. Woods, Rif S. El-Mallakh

**Affiliations:** 1 Universidade Federal de Goiás Goiânia, Goias, Brazil; 2 Federal University of São João Del Rei Campus Centro-Oeste Dona Lindú, Divinópolis, Minas Gerais Brazil; 3 School of Nursing, University of Louisville School of Medicine , Louisville, Kentucky USA; 4 Mood Disorders Research Program, Depression Center, Department of Psychiatry and Behavioral Sciences, University of Louisville School of Medicine Louisville, 401 East Chestnut Street, Suite 610, Louisville, Kentucky 40202 USA

**Keywords:** Alternating hemiplegia of childhood, *ATP1A3* gene, Bipolar disorder, Lithium, Sodium pump, Treatment

## Abstract

**Background:**

Alternating hemiplegia of childhood (AHC) shares many aspects with the psychiatric condition, bipolar disorder.

**Body:**

AHC is a heterogeneous genetic disorder that can manifest in various fashions but will usually involve paroxysmal neuropsychiatric symptoms that are precipitated by environmental events or stressors, superimposed over developmental abnormalities of various severities. It occurs as a consequence of a variety of mutations of the alpha3 subunit of the sodium pump (*ATP1A3*). Treatment is generally symptomatic with flunarizine a nonselective calcium channel blocker, but other approaches have been attempted. Nonetheless, given the inadequate response of most patients, there continues to be a significant unmet need for adequate treatment approaches. Bipolar disorder, a severe psychiatric illness, also manifests with paroxysmal neuropsychiatric symptoms that are precipitated by environmental events or stressors. It is also characterized by significant dysfunction of the sodium pump. Lithium is an effective intervention for bipolar disorder and has never been utilized in AHC.

**Conclusion:**

Both bipolar disorder and AHC are associated with sodium pump dysfunction and increased intracellular sodium concentration. Both conditions respond to calcium channel blockers and the ketogenic diet. Lithium trials in AHC are warranted.

## Introduction

Alternating hemiplegia of childhood (AHC) is a rare genetic disorder caused by various mutations of the *ATP1A3* gene. It manifests across a wide variety of severities that appear to be related to the nature of the specific mutation carried by the afflicted individual. There appear to be important similarities between pathophysiologic characteristics of AHC and bipolar disorder which suggest that AHC patients may respond favorably to effective treatments of bipolar disorder. In this review we will examine these characteristics and similarities to determine if trials with medications such as lithium may be warranted in AHC.

## Methods

This was a directed review to understand similarities between AHC and bipolar disorder. It is not intended to be systematic or complete. It is not a meta-analysis. The authors searched multiple databases as needed to address specific discussion points.

## Discussion

### AHC: clinical characteristics

Alternating hemiplegia of childhood (AHC) is a heterogeneous genetic disorder that can manifest in various fashions but will usually involve paroxysmal neuropsychiatric symptoms that are precipitated by environmental events or stressors, which are superimposed over persistent neuropsychiatric symptoms [1–3]. The paroxysmal symptoms can include variable severity of hemiplegia or quadriplegia, dystonia, nystagmus or other abnormal eye movements, autonomic dysfunction, behavioral anomalies, or seizures [1, 4, 5]. Onset is in early life and symptoms are nearly always present before age 18 months [1, 4]. The majority of patients (> 70%) with the diagnosis made on the basis of clinical symptoms [6] have various mutations of the alpha3 isoform of the sodium- and potassium-activated adenosine triphosphatase (Na, K-ATPase) or sodium pump (*ATP1A3* gene) [7]. This led to the proposal that documented *ATP1A3* mutation be one of the diagnostic criteria of the disorder [4]. However, there are other clinical syndromes that occur due to *ATP1A3* gene mutations that are seen as different from AHC. These include *r*apid-onset *d*ystonia *p*arkinsonism (RDP), *c*erebellar ataxia, *a*reflexia, *p*es cavus, *o*ptic atrophy, and *s*ensorineural hearing loss (CAPOS), *e*arly *i*nfantile *e*pileptic *e*ncephalopathy (EIEE), *c*hildhood *r*apid *o*nset *a*taxia (CROA), and *r*elapsing *e*ncephalopathy with rapid onset *a*taxia (RECA); which are collectively referred to as *ATP1A3*-related neurologic disorders [1, 8]. Nonetheless, they all share the same basic abnormalities, which include depolarization of the resting potential of neurons which increases their excitability, but reduces their ability to recover from a depolarization event. Furthermore, there are a multitude of intracellular changes which occur. These include increases of intracellular calcium [9], activation of multiple second messenger systems (in the absence of the first messenger) such as activation of transcription factors activator protein-1 (AP-1) and nuclear factor kappa-B (NF-κB) [10], as well as downstream systems including activation of the Src cascade, mitogen-activated protein kinase (MAPK), extracellular signal-regulated kinase (ERK), protein kinase B, and oroto-oncogene tyrosine-protein kinase pathways [11]. Nearly all of these changes are normalized with lithium treatment [12, 13].

Despite a large study that found that clinical course is stable for a lot of patients [14], most researchers actually find that progressive worsening, frequently with early death, is the more common course for patients [15, 16].

Behavioral or psychiatric symptoms are actually quite common in AHC, occurring in some 60–80% of patients [17–19]. In an unpublished study, onset can occur as early as < 3 years old in approximately 35% of affected individuals, but increases significantly to approximately 55% in the 4–10 years old group, and continues to increase to over 72% in adults (*P* < 0.0001). Males and females are equally affected. Psychiatric disorders diagnosed in patients with AHC spans all categories from anxiety to mood disorders, to psychosis [17–19], as well as many undiagnosed behavioral issues [20]. There are many similarities with bipolar disorder (Table 1). One interesting case is that of an *ATP1A3* mutation presenting as childhood onset ‘schizophrenia’ in a 6-year old boy [21]. Diagnosis of schizophrenia prior to the age of 13 is extremely rare, and nearly 80% of those given the diagnosis are ruled out with drug-free inpatient observation [22]. In a study of 39 patients with AHC, 64% had behavioral problems, which were mild in 2, moderate in 5, severe in 10, and extreme in 6 [19]. Extremely impacted patients generally presented with aggression, but intellectual disability was the most common issue impacting 38.5% of the patients [19]. The lack of specificity of psychiatric symptoms may simply be a reflection of the nonspecific and diffuse neuronal dysfunction of AHC or the wide variability of presenting syndromes of various mutations [18], or the lack of diagnostic rigor of psychiatric diagnoses [23]. Nonetheless, this overlap may be of value in moderating symptoms of affected individuals.

**Table 1:** Similarities between bipolar disorder and AHC

Sign or Symptom	AHC	Bipolar Disorder
Paroxysmal Neuropsychiatric Symptoms	Yes	Yes
Significant Dysfunction of the Sodium Pump	Yes	Yes
Increased Intracellular Sodium Concentration	Yes	Yes
Responds to Calcium Channel Blocker	Yes	Yes
Responds to Ketogenic Diet	Yes	Yes
Symptoms are Precipitated by Environmental Events and/or Stressors	Yes	Yes
Behavioral Abnormalities	Yes	Yes
Cognitive Dysfunction	Yes	Yes
Seizure Activity	Yes	Yes
Increased Need for Sleep	Yes	Yes (only during depressions)
Reduced Need for Sleep	No	Yes (only during manias)
Sleep Deprivation Leads to Increase of Episodes	Yes	Yes

### AHC: pathophysiology

Severity of clinical manifestations appears to be a consequence of the specific causative mutation [24]. While the diagnostic features revolve around electro-neuro-motor control, there is evidence of broad neuropsychiatric and extra-central nervous system dysfunction. Intellectual impairment occurs in the more severe cases and a psychiatric diagnosis can be made in 80% of the patients [25]. Social impairment, including autism, is not uncommon [26]. Course over time is variable. Patients’ symptoms may progress [14, 26–28], stay unchanged, or even improve [14]. But prognosis is guarded. Medical problems, such as gastrointestinal symptoms, are common and reduce quality of life [29]. Early deaths are not uncommon and may be related to pervasive cardiac abnormalities in these patients [30, 31].

AHC is rare with an estimated prevalence ranging from 1 per 100,000 [32] to 1 per 1 million children [3]. Afflicted individuals typically have no family history and usually do not reproduce, so that the maintenance of AHC in a population is probably due to *de novo* mutations [7]. Nonetheless, the fact that only three mutations account for some 60% of the cases [2] suggests hypermutable sequences in *ATP1A3* [7, 33]. The clinical manifestations of different mutations are different, but sodium pump dysfunction is demonstrable [8] with depolarization of the resting membrane potential [34].

### AHC: animal models

AHC has been modeled in animals by creating knock-in mice with human *ATP1A3* mutations. These include the Mashlool mouse line that is heterozygous for the most common human mutation, D801N [35], Myshkin mouse model of AHC, which has the I810N mutation [36], the Matoub mouse containing the E815K mutation [37], among others [38]. Myshkin mice are felt to be one of the best animal models for AHC [36], but are also reasonable animal models for bipolar disorder and both lithium and valproic acid have been found to reduce abnormal manic-like behaviors [39–41]. Homozygotes for the abnormal gene generally die at birth, usually due to respiratory failure [38]. Sodium pump expression and sodium pump activity are generally reduced in these animal models [42].

### AHC: treatment

Treatment options for AHC are quite limited. Patients will receive targeted treatment for specific manifestations, such as seizures [43] or psychiatric symptoms [25]; although there is a high rate of suboptimal response (e.g., 50% of epilepsy is treatment refractory [43], and behavioral control is frequently not successful [44]). For the core symptoms of the disease, the most commonly utilized medication in these patients is flunarizine, a nonselective calcium channel blocker [45]. The primary goal of flunarizine is to prevent, reduce the frequency, and ameliorate the motor symptoms [44]. Use of flunarizine was piloted in 1984 with a report of a single patient with a favorable response [46]. Subsequent reports, including a placebo-controlled study [47] confirmed a generally positive response. A review of some 230 patients published in 20 reports found that about half of the patients will experience a 50% drop in frequency and severity of symptoms [48]. In his review of treatments for AHC, Samanta [44] concluded that flunarizine demonstrated both efficacy and safety in AHC, but its use was limited by availability in several countries, including the United States. Verapamil, which is available in the United States, was reported to be useful in one patient in South Africa [49].

The ketogenic diet is an effective antiseizure treatment [50]. However, its use is limited because it is difficult to initiate and enforce [51]. There are several case reports of dramatic improvements in the clinical course of AHC when patients are treated with the ketogenic diet [52–55]. This has been attributed to reductions in glucose utilization as measured by positron emission tomography (PET) utilizing [^18^F]-fluorodeoxyglucose (^18^FDG) in brains of patients with AHC [56].

Topiramate is a voltage-sensitive sodium channel antagonist anti-epileptic that has had some utility particularly in patients from eastern Asia [57–59]. However, the experience of patients from Europe has been more negative [24, 27, 60]. Other voltage-sensitive sodium channel antagonists have been reported as generally negative [e.g., 59] but none of these agents has been systematically used and response appears to change based on the nature of the sodium pump mutations.

There are individual case reports of favorable response to memantine, a blocker of the glutamate-activated *N*-methyl-d-aspartate (NMDA) sodium channel [61], and aripiprazole, a dopamine D2 receptor partial agonist [62, 63]. There are also multiple reports of other interventions which are beyond the scope of our report but can be found in the review by Samanta [44].

### Bipolar disorder: clinical characteristics

Bipolar disorder is a severe psychiatric illness that is characterized by episodes of mania (comprising elevated mood and energy, grandiosity, reduced need for sleep, increased goal-directed activity, and rapid speech and thoughts) and depression (reduced mood and energy, depressed mood and low self-esteem, guilt, reduced activity, and slowed speech and thoughts) which may last weeks to months, and are interspersed between longer periods when a patient is well [64]. Episodes are frequently precipitated by environmental factors [65]. As with AHC, sleep is important, and sleep deprivation can induce episodes [64, 66]. Patients spend approximately 50% of their time asymptomatic. The other half is spent predominantly in a depressed state (approximately 40%) or manic/hypomanic state (approximately 10% [67, 68]).

Cognitive dysfunction is a common feature in bipolar disorder. While still unclear, cognitive dysfunction may be present at the time of the first manic episode, and deterioration appears to be related to severity of their mood episodes [69]. But it is not clear if the early decline in cognitive performance is due to the disease process, is a consequence of the mood episodes, or a consequence of the medications used for the treatment of the patients.

### Bipolar disorder: pathophysiology

One of the most reproduceable findings in bipolar illness is a mood-state-related reduction in sodium pump activity (both during manias and depressions [70]). This results in an increase of intracellular sodium [71, 72] with consequent change in membrane potential [73]. The reduction in sodium pump activity is intermittent and appears to be related to environmental factors [74]. Nearly all of the symptoms of bipolar disorder can be explained by the known changes in sodium pump activity [75].

### Bipolar disorder: animal models

The most commonly used animal model of mania is the amphetamine and chlordiazepoxide administration model [76, 77]. However, this model fulfills only one of the three validating criteria for an animal model (face, construct, and predictive validity [78]) [77] and is really a model for substance abuse. The intracerebroventricular administration of ouabain is the most valid pharmacologic model of bipolar disorder because it mimics biological aspects of the illness [79]. However, genetic models of reduced expression of the alpha 2 [80] and alpha 3 subunit [42] can also model mania and bipolar depression, respectively. Interestingly, these mice have been utilized to model AHC [25] and both mania and depression of bipolar illness, with adequate response to lithium and valproic acid [39–41].

### Bipolar disorder: treatment

There are two approaches to the management of bipolar disorder. The first is the use of mood stabilizing agents which act predominantly to reduce intracellular sodium and free calcium [81], and the other is to utilize agents that do not impact the abnormal pathophysiology of bipolar disorder but do result in clinical improvement, such as antipsychotic drugs [82]. A full review of treatment options for bipolar disorder is beyond the scope of our paper; instead, we will focus on treatments which appear to overlap with successful treatments in AHC.

As noted previously, flunarizine, a calcium channel blocker, is the most commonly used agent in AHC [44, 48]. Similarly, some calcium channel antagonists have been found to be useful in the treatment and prevention of mood episodes in bipolar illness, although high quality data are lacking [83, 84]. Of interest is that there is preliminary data that the ketogenic diet, which has been reported to be useful in AHC [52–55], may also be useful in bipolar disorder [85–88]. Valproic acid has documented utility in the management of bipolar disorder [89, 90]. Valproic acid has been used to treat seizures that occur in AHC but no specific effect has been reported on other neuropsychiatric symptoms [91]. Lithium is considered the best agent for the management of bipolar disorder [92] (but there are no published reports of use of lithium in AHC). Lithium may specifically have benefits in reducing intracellular sodium [93, 94] and addressing the consequences of reduced sodium pump activity [95, 96], including in Myshkin mouse model of AHC [36].

### Potential use of lithium in treatment of AHC

There are significant similarities between bipolar disorder and AHC. Both conditions are associated with dysfunction of the sodium pump and increased intracellular sodium accumulation. Increased intracellular sodium leads to increased intracellular calcium. In both conditions, there is paroxysmal worsening of the clinical state that appears to be associated with, or responsive to, environmental demands. Both conditions have some evidence of response to calcium channel blockers, valproic acid, and the ketogenic diet, but the data are not solid. The most common treatment of AHC, flunarizine, is a nonselective calcium channel blocker that reduces the effects of free calcium in excitable tissues [97]. However, the increased intracellular calcium in AHC is secondary to the increased intracellular sodium that results from the sodium pump dysfunction. Lithium reduces intracellular sodium directly in the setting of sodium pump dysfunction [93, 94].

There are also differences between bipolar disorder and AHC. Patients with AHC are more severely affected across multiple organ systems and are impacted much earlier in life. This is related to the fact that there is a clear genetic abnormality with continuous sodium pump dysfunction in AHC, while the dysfunction in bipolar disorder is paroxysmal. Exploring the specifics of these differences is important for consideration of using lithium in AHC.

Patients with AHC may have increased cardiac abnormalities. Nearly 70% of patients with the D801N variant will have shortened QT_c_ with associated increased risk of ventricular arrhythmias and cardiac arrest [98, 99]. Lithium may exhibit opposing cardiac effects [100–102]; at therapeutic levels lithium appears to be cardioprotective in humans and animals [103, 104], while at toxic levels it may predispose to arrhythmia [105]. Importantly for consideration in AHC, lithium does not alter the QT_c_ interval [105]. The cardioprotective effect of lithium appears to occur even at very low lithium consumption [102, 106, 107].

AHC is frequently associated with muscle involvement with symptoms that include plegia (weakness), altered muscle tone with either increased tone (dystonia) or reduced tone (hypotonia), and movement disorders such as choreoathetosis and ataxia [108, 109]. Patients with bipolar disorder do not have motor involvement as part of their condition, but may be more susceptible to developing movement disorders secondary to psychiatric medications [110] and at a higher risk of developing neurologic conditions such as Parkinson’s disease [111]. Lithium may induce tremor with typical treatment [112]. Because lithium is frequently coadministered with an antipsychotic [113, 114], side effects of antipsychotics, such as parkinsonism or dystonia, are frequently attributed to lithium. In fact, in long term studies, lithium actually protects against movement disorders [115].

Patients with AHC will frequently have unanticipated complications when receiving anesthesia [116–118]. Complications are most common among patients with *ATP1A3*-related disease, and among those, ones with the D801N variant have the most severe complications [117, 118]. Patients with bipolar disorder may also have complications with surgery that are milder and occur independently of medications being prescribed [119, 120]. However, the use of lithium increases interactions with many anesthetic agents, and particularly with neuromuscular blockers [121]. For this reason, lithium is usually held prior to general surgery, although despite a higher rate of problems [122] it is frequently continued when patients receive anesthesia for electroconvulsive therapy (ECT) [123]. If lithium is used in AHC, discontinuation prior to surgery would likely become the standard.

Adenosine triphosphate (ATP) is the fuel that power the sodium pump, but is also utilized as a modulator of brain activity [124]. Dysregulation of extracellular ATP concentrations appears to be a general marker of cellular dysfunction and is believed to play a role in AHC [125, 126], a wide array of psychiatric conditions [124, 127, 128], and non-brain conditions such as cardiac ischemia [129]. The nonspecificity suggests that ATP concentration dysregulation is just another marker that “something is wrong” like inflammatory markers, and does not represent a pathoetiologic clue to these conditions [130]. Nonetheless, administration of oral ATP supplementation may be of benefit in both AHC and psychiatric conditions [125, 126, 131].

## Conclusion

There are significant similarities between bipolar disorder and AHC. Both conditions are associated with dysfunction of the sodium pump and increased intracellular sodium accumulation. In both conditions, there is paroxysmal worsening of the clinical state that appears to be associated with, or responsive to, environmental demands. Both conditions have some evidence of response to calcium channel blockers, valproic acid, and the ketogenic diet, but the data are not solid. Nonetheless, given the similarities between the two conditions, documented response to lithium in animal models of both conditions, and the known actions of lithium on sodium transport, it is reasonable to expect that lithium may be effective in AHC.

Nonetheless, it is important to remember that lithium is not a benign medication and, with the availability of alternative treatments, the use of lithium in bipolar disorder has significantly dropped despite data that show its superiority [132]. Thus, research into the utility of lithium in AHC needs to proceed with reasonable caution and awareness, with adequate attention to the balance between potential harm and potential benefit.

With this caveat, trials of lithium in mitigation of neuropsychiatric symptoms experienced by patients afflicted with AHC are warranted, particularly given the unmet need of inadequate treatments in this disorder.

## Data Availability

Not applicable.

## Abbreviations

AHC: Alternating Hemiplegia of Childhood
CAPOS: Cerebellar ataxia, Areflexia, Pes cavus, Optic atrophy, and Sensorineural hearing loss
CROA: Childhood Rapid Onset Ataxia
EIEE: Early Infantile Epileptic Encephalopathy
Na,K-ATPase: Sodium- and Potassium-Activated Adenosine Priphosphatase
PET: Positron Emission Tomography
RDP: Rapid-onset Dystonia Parkinsonism
RECA: Relapsing Encephalopathy with Rapid onset Ataxia
